# A method for lipoprotein (a) Isolation from a small volume of plasma with applications for clinical research

**DOI:** 10.1038/s41598-022-13040-4

**Published:** 2022-06-01

**Authors:** Paul A. Mueller, Elisabeth Yerkes, Paige Bergstrom, Sara Rosario, Joshua Hay, Nathalie Pamir

**Affiliations:** Center for Preventive Cardiology, Knight Cardiovascular Institute, Oregon Health & Science University, 3161 SW Pavilion Loop, Mail Code UHN62, Portland, OR 97239 USA

**Keywords:** Isolation, separation and purification, Dyslipidaemias, Atherosclerosis

## Abstract

High levels of circulating Lipoprotein (a) [Lp(a)] are an independent risk factor for CVD. One of the major limitations to investigating Lp(a) biology is the need for large volumes of plasma (4–10 mL) for its isolation. We developed an isolation technique requiring only 0.4 mL of plasma yielding an enriched Lp(a) fraction suitable for compositional and functional studies. We collected plasma from patients (n = 9) in EDTA presenting to our Center for Preventive Cardiology for CVD risk management and with circulating Lp(a) > 66 mg/dL. 0.4 mL of plasma was added to 90 µL of potassium bromide (1.33 g/mL) and subjected to our two-step density-gradient ultracentrifugation method. The first step separates VLDL and LDL from the Lp(a) and HDL fractions and the second step further separates VLDL from LDL and Lp(a) from HDL. Lp(a) is then dialyzed for up to 24 h in potassium phosphate buffer. We performed cholesterol gel electrophoresis, immunoblotting and LC-MS/MS proteomics on isolated lipoprotein fractions to confirm fraction enrichment. Functional studies including Lp(a)-dependent induction of macrophage gene expression and cholesterol efflux inhibition were performed on isolated Lp(a) to confirm its preserved bioactivity. Lp(a) yields (264 ± 82.3 µg/mL on average) correlated with Lp(a) plasma concentrations (r^2^ = 0.75; p < 0.01) and represented the relative distribution of circulating apo(a) isoforms. Proteomic analyses confirm lipoprotein fraction separation. Functional integrity was confirmed by the findings that isolated Lp(a) inhibited plasminogen-dependent cholesterol efflux in HEK293T cells expressing ABCA1 and increased expressions of *Il1b*, *Nos2* and *Ccl2*. We developed a small-volume isolation technique for Lp(a) suited for a range of applications used in biomedical research. The use of this technique circumvents volume-dependent limitations and expands our ability to investigate the mysteries of this deleterious lipoprotein.

## Introduction

Clinical studies have identified elevated levels of lipoprotein (a) [Lp(a)] as an independent risk factor for coronary artery disease (CAD)^1–3^. Lp(a) shares structural similarities with LDL, but contains an additional protein, apolipoprotein (a) [LPA], covalently bound to apolipoprotein B (APOB) in a 1:1 ratio^1^. LPA is encoded by the *LPA* gene and evolved as a duplication of the plasminogen gene^4,5^. Polymorphisms in *LPA* result in a variable number of plasminogen kringle IV type-2 (KIV-2) domain repeats (ranging from 3 to 43), with a resulting wide variation in size (between 400 and 800 kDa)^6^. Of note, LPA size is negatively correlated with Lp(a) plasma concentration^7^.

Though the mechanisms behind Lp(a)-mediated increased CVD risk are not defined, it is known that: 1. Lp(a) impedes the fibrinolytic cascade by inhibiting plasminogen activation by the proteolytic enzyme, t-PA (tissue plasminogen activator)^8^; 2. Lp(a) inhibits plasminogen-mediated, ABCA1-dependent cholesterol efflux capacity in macrophages^9,10^; 3. Similar to LDL, Lp(a) accumulates within the subintimal space of the artery wall resulting in increased intravascular cholesterol and inflammation^11,12^.

Statins do not lower Lp(a) levels^13–16^, whereas PCSK9 inhibitors reduce Lp(a) levels on average by 25–30%^17^. Currently, lipoprotein apheresis is the only clinically reliable method for Lp(a) lowering^18,19^ and methods to silence LPA expression are showing promise in clinical trials^20^. The lack of effective treatments stems in part from a deep gap in our understanding of Lp(a) biology, mostly due to a lack of animal models. Mice, the most common animal model used to study atherosclerosis, do not carry an *LPA* ortholog and therefore have no circulating Lp(a)^21^. Thus, the most available avenue for studying Lp(a) is to use freshly isolated lipoprotein from human donors or non-human primates in in vitro settings. However, even isolating Lp(a) proves difficult as it requires between 4 and 10 mL of plasma^22,23^. von Zychlinski et al. have previously combined density gradient ultracentrifugation with size exclusion-fast flow liquid chromatography (FPLC) to isolate purified Lp(a) sufficient to characterize the lipoprotein’s proteome using mass spectrometry^22^. While this method is sufficient to generate purified Lp(a) for mass spectrometry, the use of FPLC causes to sample to be highly diluted, an important limitation for subsequent functional studies. The labor- and time-intensive nature of size exclusion chromatography limits the value for cohort-based clinical research, which requires scalable high-throughput approaches.

Herein, we describe a method for the reproducible isolation and characterization of Lp(a) from plasma using sequential density-gradient ultracentrifugation, requiring only 0.4 mL, and additionally yielding VLDL and LDL as well. Using liquid chromatography tandem mass spectrometry (LC-MS/MS), we characterize the Lp(a) proteome and compare it to VLDL and LDL from the same isolation as well as HDL from an independent isolation of the same plasma batch. We also investigate whether isolated Lp(a) preserves its functional integrity by measuring Lp(a)-dependent induction of macrophage gene expression and cholesterol efflux. The ability to isolate Lp(a) fractions from small plasma volumes using a commonly available ultracentrifugation method will facilitate studies of this complex and understudied lipoprotein in clinical cohorts.

## Materials and methods

### Sample collection

**Figure 1:**
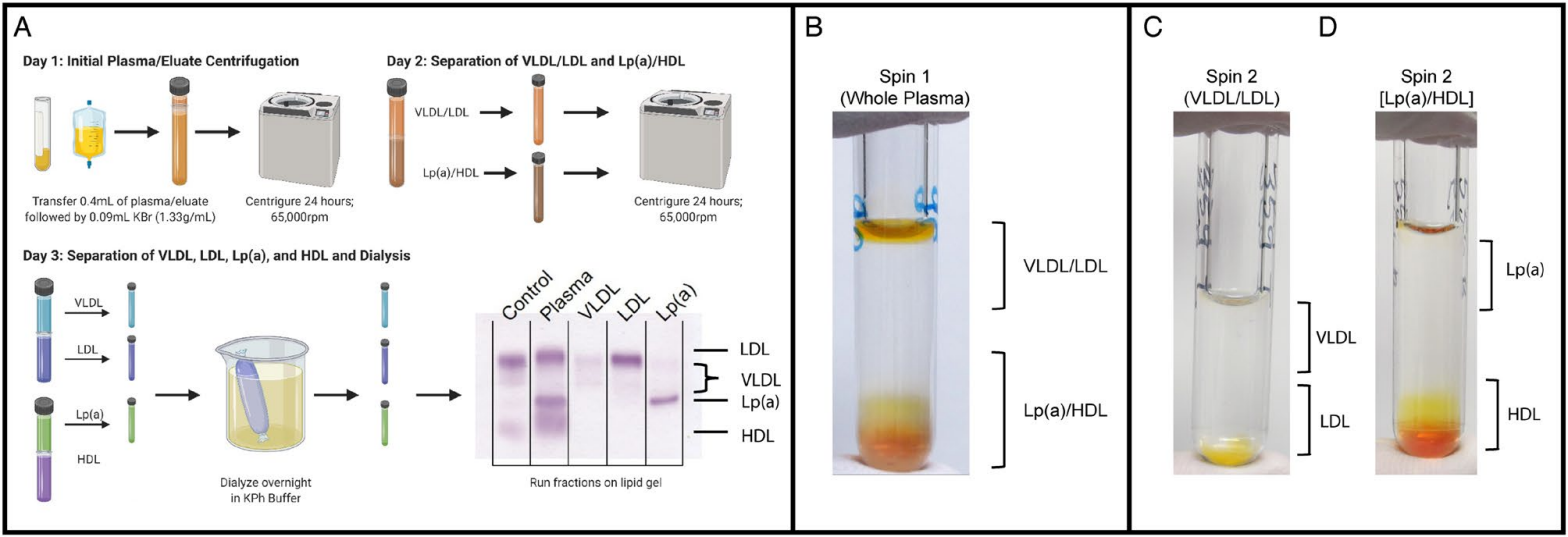
Small-volume lipoprotein isolation workflow. (**A**) Workflow for small-volume isolation of Lp(a), VLDL and LDL from whole plasma. HDL is isolated using a separate ultracentrifugation method described in Materials and Methods but is included in this diagram for visualization. (**B**) Example image of lipoprotein separation following the first ultra-centrifugation spin. VLDL/LDL fractions are found at the top of the suspension while Lp(a)/HDL localize to the bottom. (**C**) Example image of the separation of VLDL and LDL following the second ultra-centrifugation spin. (**D)** Example image of the separation of Lp(a) and HDL following the second ultra-centrifugation spin. Figure 1 was generated using www.BioRender.com .

This study derives from a prospectively designed clinical cohort in which all subjects were enrolled and informed consent was obtained by the registry and biorepository of the Center for Preventive Cardiology (CPC) at OHSU^24^ and conducted under the approval of the OHSU Institutional Review Board (#STUDY00017329). In addition, this study abides by the Declaration of Helsinki principles. Whole blood for lipoprotein fraction isolation and characterization was collected from patients with circulating Lp(a) > 50 mg/dL. Blood samples were drawn during their initial visit and plasma lipid panel including Lp(a) concentrations were measured as previously described^24^. Whole blood was collected into EDTA-tubes and 0.4 mL of plasma was subjected to small volume isolation of VLDL, LDL and Lp(a) using density gradient ultra-centrifugation as described in the workflow presented in Fig. 1A. An additional 0.1 mL of plasma was used for the isolation of HDL using ultra-centrifugation as previously described^25^.

### Mice

Animal care and all experimental procedures were performed according to the regulation of OHSU’s Institutional Animal Care and Usage Committee (IACUC; Protocol# IP00002733). The OHSU IACUC is accredited by the AAALAC International and in compliance with Animal Welfare Act regulations and Public Health Service Policy. This study was performed and reported in accordance with the ARRIVE guidelines.

### Small volume lipoprotein fraction isolation

#### Materials

##### Sequential gradient ultracentrifugation

1. Potassium bromide (KBr) solutions at density of 1.33 g/mL and 1.09 g/mL (Millipore Sigma Cat# 449,962)
2. Optima MAX-XP tabletop ultracentrifuge (Beckman Coulter Cat# 393,315)
3. TLA-100.3 fixed-angle rotor (Beckman Coulter Cat# 349,481)
4. 0.5 mL Open-top thick wall polycarbonate tube, 8 × 34 mm (Beckman Coulter Cat# 362,224)
5. 250µL Hamilton syringe with Chaney adapter (Millipore Sigma Cat# 24,542)
6. BD PrecisionGlide needle, 21 G × 1.5 inch (BD Biosciences #305,167)
7. Normal saline: 0.9% sodium chloride and 0.5 mM ethylenediaminetetraacetic acid (EDTA)
8. Potassium phosphate dialysis buffer: 14.77 mM dibasic KPh, 5.23 mM monobasic KPh, 101.4 μM diethylenetriaminepentaacedtic acid (DTPA)
9. Slide-A-Lyzer™ MINI Dialysis Device, 3.5 K MWCO, 0.1 mL (ThermoFisher #69,552).
10. Magnetic stirring hot plate
11. Floating foam tube rack
12. Kimtech Science Kimwipes Delicate Task Wipers, 1-Ply (Fisher Scientific Cat# 06-666C)
13. 1.5 mL Eppendorf Tubes
14. Pierce BCA Protein Assay Kit (Thermo Fisher Scientific Cat# 23,225)

##### Cholesterol gel electrophoresis for visualization of lipoproteins

1. QuickGel Chamber for cholesterol gel electrophoresis (Helena Labs #1284)
2. QuickGel Cholesterol Profile Kit (Helena Labs #3543 T)

##### Lipoprotein cholesterol analysis

1. Wako cholesterol E assay (Wako Diagnostics #999-02,601)
2. Control serum I (Wako Diagnostics #410-00,102)
3. Control serum II (Wako Diagnostics #416-00,202)
4. Microplate spectrophotometer (Thermo Fisher Scientific or any other brand)

#### Methods

A workflow is provided in Fig. 1A for reference.

##### Day 1: sample mixing and preparation.

1. Frozen plasma samples are thawed for 15 min in a beaker of 37ºC water bath.
2. While not included in this study, this method can be used to isolate Lp(a) from lipoprotein apheresis eluate, a therapeutic method of removing APOB containing lipoproteins such as LDL, Lp(a) and VLDL, from plasma. If using eluate from plasma apheresis, filter using a 100 µm cell straining kit into a 50 mL conical tube to remove debris.
3. Transfer 0.4 mL of plasma or eluate to a 0.5 mL Open-top thick wall polycarbonate ultracentrifuge tube followed by 90µL of KBr (1.33 g/mL) for a final density of 1.066 g/mL.
4. Tubes should be balanced to within 0.02 g.

##### Centrifugation

5. Samples are loaded into the TLA 100.3 fixed-angle rotor and centrifuged in the Optima MAX-XP tabletop ultracentrifuge at 65,000 rpm at 4 °C for 24 h.

##### Day 2: fraction collection

1. Following the 24-h ultracentrifugation there are two distinguishable layers: the top layer composed of VLDL and LDL; and the bottom layer composed of HDL and Lp(a) (Fig. 1B).
2. 125µL of the VLDL/LDL top layer is carefully collected and transferred to a fresh labeled 0.5 mL Open-top thickwall polycarbonate tube.
3. Next, 300-350µL of the Lp(a)/HDL layer is collected by pipetting from the very bottom of the tube being careful not to draw up the last few microliters of the volume which will be contaminated with LDL. Lp(a)/HDL layer is then transferred to a fresh labeled 0.5 mL Open-top thickwall polycarbonate tube.
4. Add 216µL of normal saline was to the VLDL/LDL centrifuge tube for a final density of 1.027 g/mL.
5. To the Lp(a)/HDL centrifuge tube, add 20µL of KBr (1.33 g/mL) and 115µL of KBr (1.09 g/mL) for a final density of 1.082 g/mL.

##### Centrifugation

6. VLDL/LDL and Lp(a)/HDL solutions are loaded into the TLA 100.3 fixed-angle rotor and centrifuged in the Optima MAX-XP tabletop ultracentrifuge at 65,000 rpm at 4 °C for 24 h.

##### Day 3: fraction collection and dialysis

1. Pre-soak dialysis cups by adding ~ 0.3–0.5mLs potassium phosphate buffer and letting them sit suspended in a tube rack for 10 min prior to use. Inspect for leaks or malfunction using the manufacturer’s instructions for reference.
2. Each ultracentrifuge tube will now be separated into two layers. The upper layer of the VLDL/LDL tube is comprised of the VLDL fraction, and the bottom layer will be the LDL fraction (Fig. 1C). The upper layer of the Lp(a)/HDL tube is comprised of Lp(a) and the bottom layer contains the HDL fraction along with all other plasma proteins (Fig. 1D). Use a Hamilton syringe to carefully collect the 100uL from each layer (except for the HDL layer) and transfer the collection directly onto the center of the Slide-A-Lyzer™ MINI Dialysis Device membrane, avoiding the sides of the cup. Take care to draw volume from the very top of the tubes for VLDL and for Lp(a) and from the very bottom of the centrifuge tube when collecting the LDL layer.
3. Load dialysis device cups into a floating foam tube rack and dialyze overnight at 4 °C in 1.5–2.0L potassium phosphate dialysis buffer. Dialysis should be performed on a magnetic stirring hot plate with a magnetic bar continuously stirring the potassium phosphate dialysis buffer.

##### Day 4: dialysis

1. The following morning, discard the potassium phosphate dialysis buffer. Replace dialysis cups on the floating foam tube racks in fresh potassium phosphate dialysis buffer for 3 h at 4 °C.
2. Once more, discard the potassium phosphate dialysis buffer. Replace dialysis cups on the floating foam tube racks in fresh potassium phosphate dialysis buffer for another 3 h at 4 °C.
3. Discard the potassium phosphate dialysis buffer and tap the dialysis cups against a Kimwipe wiper to remove any additional dialysis buffer. Centrifuge the dialysis cups in 1.5 mL Eppendorf tubes at 14,000 g for 20 min at 4 °C to burst the membrane and collect sample.
4. Total lipoprotein concentrations and yield are determined using BCA protein assay per the manufacturer’s instructions. % Recovery was calculated by determining the expected yield in each lipoprotein fraction from 0.4 mL of plasma for VLDL, LDL, and Lp(a) and from 0.1 mL of plasma for HDL based on the subjects’ initial plasma concentrations (determined using Roche Hitachi 704 Chemistry Analyzer). Total yield is then divided by the expected yield for each fraction and multiplied by 100.
5. Lipoprotein fractions are subjected to size separation by cholesterol gel electrophoresis using QuickGel Cholesterol Profile kit to confirm enrichment of the isolation according to the manufacturer’s instructions.
6. To confirm lipoprotein fraction enrichment and visualize cholesterol distribution, 100 µL of isolated VLDL, LDL, Lp(a), and HDL were injected into a Superose 6 Increase 10/300 GL size-exclusion liquid chromatography column. Total cholesterol in each fraction is determined with Pointe Scientific Cholesterol Liquid Reagent Set per the manufacturer’s instructions.

## HDL sample preparation and ultracentrifugation

HDL was isolated by a two-step density gradient ultracentrifugation method by layering the density-adjusted plasma (1.24 g/ml) underneath a NaCl-density solution (1.006 g/ml) as previously described^25,26^. Tubes were sealed and centrifuged at 65,000 rpm for 6 h in a 90Ti fixed angle rotor (Beckman Instruments, Krefeld, Germany). After centrifugation, the HDL-containing band was collected, desalted via PD10 columns (GE Healthcare, Vienna, Austria) and immediately used for experiments.

### Lipoprotein digestion

Lipoprotein fraction protein concentration was determined using the bicinchoninic assay (BCA) with bovine serum albumin as the standard. Lipoprotein fractions [VLDL, LDL, Lp(a), and HDL] were digested as previously described^31,38^. Each fraction (10 µg protein) was solubilized with 0.1% RapiGest (Waters) in 100 mM ammonium bicarbonate, reduced with dithiothreitol, alkylated with iodoacetamide, and digested with trypsin at a 1:10 protease:protein ratio (Promega, Madison, WI) overnight at 37 °C. After acidic hydrolysis of RapiGest with 0.5% trifluoroacetic acid, samples were dried, and stored at − 20 °C until MS analysis.

### LC-MS/MS

Tryptic digested lipoprotein samples were reconstituted in 5% acetonitrile and 0.1% formic acid. Isolated lipoprotein tryptic digests (1 µg) were separated by a 90-min linear gradient of solvents A (0.1% formic acid in water) and B (0.1% formic acid in acetonitrile) with a nanoACQUITY UPLC with an Acclaim PepMap nanoLC C18, 75 µm x 25 cm column. Data-dependent acquisition (DDA) LC–MS/MS proteomics was performed on ultracentrifugally isolated lipoprotein fractions. Lipoprotein fractions (10 µg) were subjected to tryptic digestion as described above. Before analysis, DDA by Top 10 method was performed on a Q Exactive HF.

### Proteome analysis

RAW instrument files were processed using Proteome Discoverer version 1.4.1 (Thermo Scientific) using SEQUEST HT software and a human protein canonical database downloaded in January 2019 from the UniProt Sprot server. Searches were configured with static modification for carbamidomethyl (+ 57.021 Da) on cysteines, dynamic modifications for oxidation of methionine residues (+ 15.9949 Da), parent ion tolerance of 1.25 Da, fragment mass tolerance of 1.0005 Da, and monoisotopic masses. Only peptides with q scores < 0.05 were accepted, and at least 2 unique peptides had to be matched to a protein entry for its identification. Protein relative abundance was determined by normalizing peptide spectral matches (PSMs) to total PSMs^26,27^. To characterize proteins as being lipoprotein-specific, the original protein list generated from DDA LC-MS/MS proteomics was filtered to exclude any proteins that did not appear in ≥ 75% of samples from at least one lipoprotein compartment.

### In vitro analysis of gene expression in macrophages

Peritoneal macrophages were collected from C57BL6/J mice (Jackson Laboratory) 4 days after intraperitoneal injection with 3% thioglycolate broth and seeded into 12-well plates at 1 × 10^6^ cells per well with DMEM (Gibco) containing 10% FBS and 1% Penstrep (Gibco) as previously described^28^. To generate foam cell macrophages, macrophage cell media was switched to DMEM with 0.1% fatty acid-free bovine serum albumin containing 2% serum previously collected from *Ldlr*^-/-^ mice (Jackson Laboratory) on Western-type diet (Envigo) for 12 weeks^9^. Macrophages were incubated for 24 h in *Ldlr*^-/-^ serum before being treated with vehicle or previously frozen isolated Lp(a) (15 µg/mL) for 4 h. Lp(a) was derived from our small-volume isolation method and performed using Lp(a) from 4 separate subjects. Cells were then washed 3 × in ice cold 1X PBS and RNA extracted using E.Z.N.A. Total RNA Kit 1 (Omega Bio-Tek) according to the manufacturer’s instructions. cDNA was generated from 1 µg of RNA using iScript cDNA Synthesis Kit (Bio-Rad) and gene expression quantified via qRT-PCR using the Applied Biosystems ViiA 7 Real-Time PCR System. Taqman primers targeting murine *Il1b*, *Nos2* and *Ccl2* were obtained from ThermoFisher Scientific.

### In vitro cholesterol efflux analysis of ABCA1-transfected HEK293T cells

The plasmid constructs expressing human *ABCA1* was prepared by cloning human full-length cDNA into pcDNA3.1( +) mammalian expression vector, and the cDNA sequence was confirmed by DNA sequencing. HEK293T cells were transiently transfected with ViaFect Transfection Reagent (Promega) at 37 °C overnight. Once transfected, cholesterol efflux capacity was measured as previously described^9,29^. Briefly, ABCA1-expressing HEK293T cells were radiolabeled with 2µCu [^3^H]cholesterol overnight at 37 °C. Efflux of [^3^H]cholesterol was measured after a 4-h incubation in media only, 6 µg/mL of previously frozen Lp(a), 2µg/mL plasminogen, and 6µg/mL Lp(a) pretreated for 10-min followed by 2µg/mL plasminogen. Lp(a) was derived from our small-volume isolation method and performed using Lp(a) from 4 separate subjects. Cholesterol efflux was calculated as the percentage of radiolabel in the medium of the cells at the end of the 4-h incubation period divided by the total radioactivity of the medium and cells. Percent efflux is then presented as fold-change relative to media only controls.

### Statistical analyses

A Pearson’s Correlation was used to assess the relationship between subjects’ plasma Lp(a) concentrations and their respective Lp(a) yield following our small volume isolation method. For comparisons between specific lipoprotein groups, Levene’s test for homogeneity of group variances was performed for each protein to verify heteroscedasticity, where F > 0.05 was considered significant. Significant changes in protein abundance between groups was determined by an unpaired two-sided t-test, followed by Benjamini–Hochberg correction. Log_2_ fold changes in protein abundance and their -log_10_ adjusted P-values are displayed using volcano plots. A differentially expressed protein was selected by having greater than onefold change and adjusted *P* value < 0.05. A principal component analysis (PCA) was conducted, and score plots produced. Distinct proteomic signatures of each lipoprotein group were visualized with heat maps, where relative protein abundance was calculated as Z-scores generated from adjusted spectral counts. Samples and proteins were clustered by complete Euclidian hierarchical cluster analyses (agglomerative clustering) with complete sub-data sets^26,27^.

### Data and software availability

The mass spectrometry proteomics datasets produced in this study have been deposited to the ProteomeXchange Consortium via the PRoteomics IDEntifications (PRIDE)^30^ partner repository with the dataset identifier: PXD023432^30^. A github page that contains source files and R analyses is found at https://github.com/phbergstrom/Lpa-small-volume-isolation-proteomics.

## Results

### Lipoprotein fraction isolation

The patient cohort consisted of 3 males and 4 females presenting with an Lp(a) range of 66 to 282 mg/dL and a median concentration of 162 mg/dL (IQR 93.5 mg/dL; Table 1A and B). Mean HDL-C and LDL-C were 55.9 ± 15.6 mg/dL and 117 ± 59.0 mg/dL, respectively (Table 1B).

**Table 1:** Patient Characteristics and Lipid Profiles.

Variable	(N = 7)
A. Demographic information	
**Sex-no. (%)**	
Male/Female	3 (43%) / 4 (57%)
**Age at blood collection-yr**	
Mean [(SD) (Min–Max)]	67.4 [(6.3) (5–75)]
**BMI**	
Mean [(SD) (Min–Max)]	30.1 [(5.2) (21.–36.5)]
B. Lipid Characteristics	
**Total cholesterol-mg/dL**	
Mean [(SD) (Min–Max)]	199 [(58.9) (114–283)]
**Total triglycerides-mg/dL**	
Mean [(SD) (Mi–Max)]	133 [(74.1) (4–262)]
**High density lipoprotein cholesterol-mg/dL**	
Mean [(SD) (Min–Max)]	55.9 [(15.6) (38–79)]
**Low density lipoprotein cholesterol-mg/dL**	
Mean [(SD) (Min–Max)]	117 [(59.0) (38–197)]
**Very low density lipoprotein cholesterol-C-mg/dL**	
Mean [(SD) (Min–Max)]	26.6 [(14.6) (9–52)]
**Lipoprotein(a)-mg/dL**	
Median [(IQR) (Min–Max)]	162 [(93.5) (66–282)]

**Figure 2:**
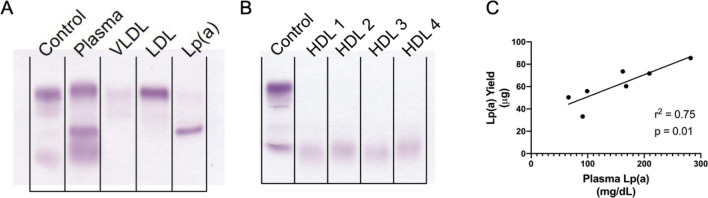
Lipid gel analysis demonstrates lipoprotein fraction purity following small-volume isolation. 0.4 mL of patient plasma was subjected to a two-step density gradient ultracentrifugation using potassium bromide. An additional 0.1 mL of plasma was subjected to a separate density gradient ultracentrifugation to isolate HDL. Isolated lipoprotein fractions were then subjected to dialysis in potassium phosphate buffer. (**A**) Dialyzed VLDL, LDL and Lp(a) lipoprotein fractions were run on QuickGel Cholesterol Profile Kit lipid gels to determine fraction purity. (**B**) Similarly, HDL was run on QuickGel Cholesterol Profile Kit lipid gels. (**C**) Average lipoprotein yields (µg) from each patient were determined using BCA assay and Pearson’s correlation was performed with known plasma Lp(a) concentrations (mg/dL).

VLDL, LDL, and Lp(a), were isolated from 0.4 mL of plasma using the small-volume isolation method described in Methods and outlined in Fig. 1. HDL was isolated using 0.1 mL of plasma in parallel with an established ultracentrifugation method^25^. Isolated lipoprotein fraction profiles were determined using QuickGel Cholesterol gels and shown in Fig. 2A and B. Each isolated lipoprotein fraction was subjected to Western blotting to visualize APOB, LPA, APOA1, and APOE (Supplemental Fig. 1). As expected, APOB was present in VLDL, LDL, and Lp(a), but not HDL (Supplemental Fig. 1A). LPA was present in Lp(a) as well as LDL (Supplemental Fig. 1B). APOA1 was abundant in Lp(a) and HDL, and minimally detected in some LDL (Supplemental Fig. 1C). APOE was observed in every lipoprotein fraction isolated (Supplemental Fig. 1D). Isolated fractions were then subjected to FPLC to determine cholesterol distribution (Supplemental Fig. 1E). We observed a single distinctive cholesterol peak for LDL and HDL, and two for Lp(a). VLDL was not detected likely due to its low yield.

Lipoprotein fraction concentrations were determined with BCA protein assay and summarized in Table 2. Small-volume isolation of Lp(a) yielded an average of 61.5 ± 17.2 µg over 2 to 4 isolations per subject (average concentration 263.9 ± 80.0 µg/mL and average % recovery of 11.2 ± 4.08%) and resolved as a distinct band on the cholesterol gel (Fig. 2A, final lane). The average LDL isolation yielded 138.9 ± 68.3 µg. As expected, based on its low abundance in fasting plasma, VLDL lipoprotein represented the lowest yields and averaged 49 ± 19.8 µg. Lp(a) isolation yields were significantly correlated with plasma Lp(a) levels (r^2^ = 0.75; p = 0.01) (Fig. 2C). Because Lp(a) size and density overlap with that of LDL^22^, we evaluated the presence of LPA in the LDL fraction and determined that 93.9 ± 3.31% of LPA protein resides in the isolated Lp(a) fraction (Supplemental Fig. 2; the whole blot is presented in Supplemental Fig. 5).

**Table 2:** Small-volume lipoprotein isolation yields. Lipoprotein yields and concentrations are determined by Pierce BCA assay and are displayed as average ± SD from 2 to 4 individual isolations for each subject.

Lp(a)						LDL					VLDL				
Sample ID	Plasma Concentration (mg/dL)	Fraction Concentration (μg/mL) ± SD	Total Yield (μg) ± SD	Total Yield CV	Percent Recovery (%)	Plasma Concentration (mg/dL)	Fraction Concentration (μg/mL) ± SD	Total Yield (μg) ± SD	Total Yield CV	Percent Recovery (%)	Plasma Concentration (mg/dL)	Fraction Concentration (μg/mL) ± SD	Total YIELD (μg) ± SD	Total Yield CV	Percent Recovery (%)
354	66	207.9 ± 51.7	50.4 ± 7.13	14.2	19.1	197	800.8 ± 86.7	205.5 ± 13.9	6.75	26.08	27	247.2 ± 52.6	60.9 ± 8.99	14.76	22.6
359	99	222.3 ± 52.0	55.9 ± 13.0	23.3	14.1	74	458.1 ± 22.7	120.8 ± 8.18	6.77	40.82	15	157.3 ± 26.8	32.9 ± 11.8	35.79	22.0
369	168	223.5 ± 23.7	60.3 ± 7.74	12.8	8.97	116	323.3 ± 18.0	84.0 ± 10.7	12.7	18.1	21	230.3 ± 43.0	62.5 ± 5.23	8.4	29.78
392	282	420.0 ± 170.6	85.5 ± 18.64	21.8	7.58	93	434.2 ± 79.5	116.1 ± 17.5	15.1	31.2	23	134.2 ± 27.9	35.8 ± 7.99	22.3	15.6
413	91	178.5 ± 112.7	33.2 ± 8.91	26.8	9.12	110	577.8 ± 110.9	147.6 ± 7.35	4.98	33.55	39	235.2 ± 14.8	63.7 ± 1.56	2.44	16.33
422	162	286.5 ± 82.3	73.5 ± 27.82	37.8	11.3	193	952.0 ± 77.4	247.5 ± 36.6	14.8	32.1	52	309.2 ± 12.9	71.9 ± 9.13	12.7	13.8
556	209	308.3 ± 66.4	71.7 ± 5.58	7.77	8.58	38	213.4 ± 44.7	50.4 ± 4.39	8.70	33.16	9	74.6 ± 41.8	19.6 ± 11.4	58.08	21.7
Average	153.9 ± 75.7	263.9 ± 82.3	61.5 ± 17.2	20.6 ± 10.1	11.2 ± 4.08	117.3 ± 59.0	537.1 ± 261	138.8 ± 68.3	9.97 ± 4.17	30.7 ± 7.05	26.6 ± 14.6	166.9 ± 98.4	49.61 ± 19.8	22.1 ± 19.1	20.2 ± 5.48

To test the lower limitations of this small-volume isolation technique, we isolated Lp(a) from two additional subjects from our clinical cohort with circulating Lp(a) concentrations < 50 mg/dL. The percent yield in subjects with Lp(a) < 50 mg/dL was similar to those with Lp(a) ≥ 66 mg/dL and was 11.4% and 12.7% in subjects with plasma Lp(a) of 30 mg/dL and 11 mg/dL, respectively (Supplemental Table 1). The actual Lp(a) yield was 13.7 and 5.6µg in subjects with 30 and 11 mg/dL, respectively.

### Proteome characterization of small-volume isolated lipoprotein fractions

**Figure 3:**
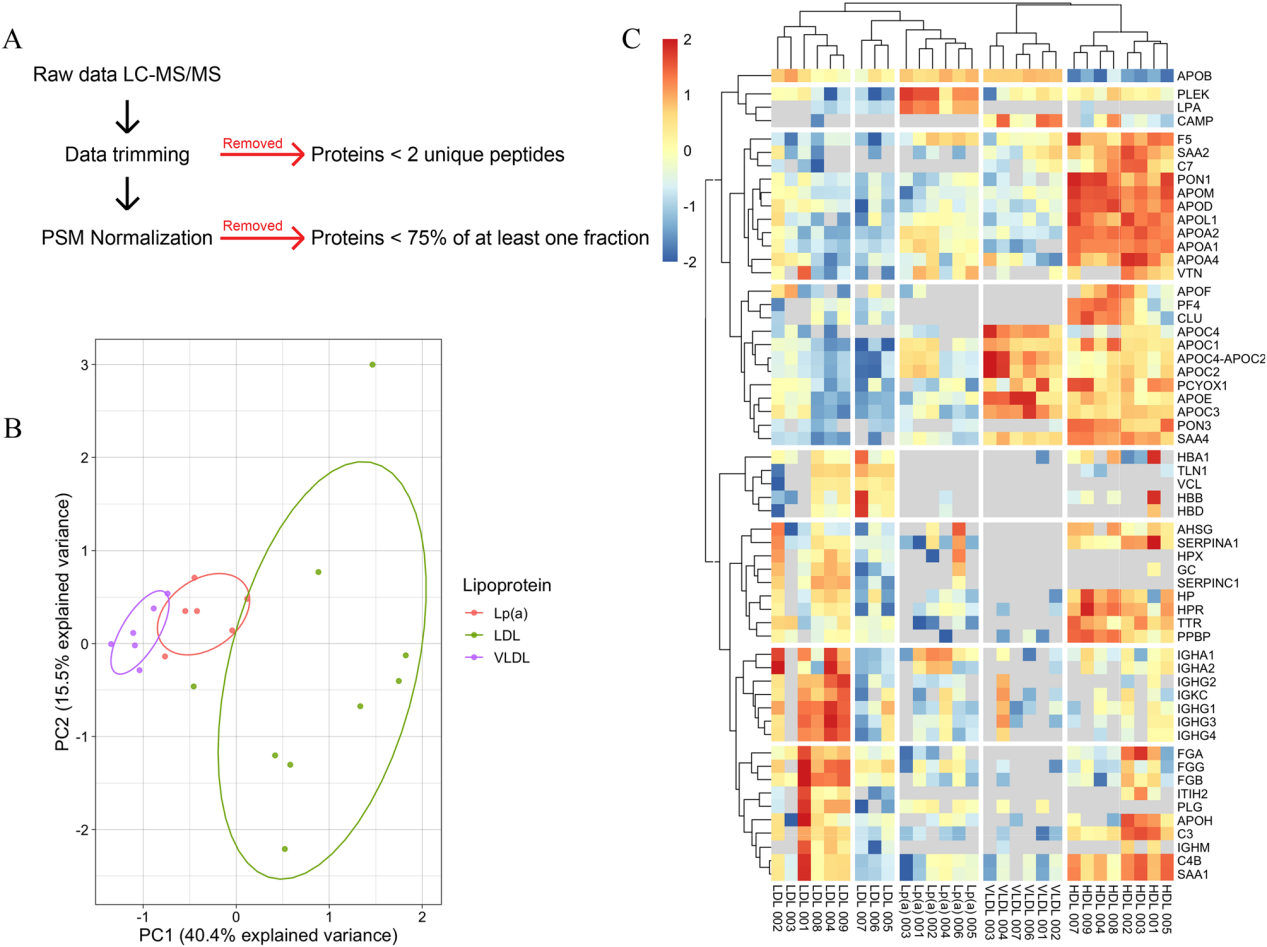
Proteomic characterization of isolated lipoprotein fractions from patients with elevated Lp(a). Lipoprotein fractions were isolated from whole plasma using a two-step density gradient ultracentrifugation and then dialyzed in potassium phosphate buffer. Isolated lipoprotein tryptic digests (1 µg) were separated by a 90-min linear gradient of solvents A (0.1% formic acid in water) and B (0.1% formic acid in acetonitrile) with a nanoACQUITY UPLC with an Acclaim PepMap nanoLC C18, 75 µm x 25 cm column. Data-dependent acquisition by Top 10 method was performed on a Q Exactive HF. (**A**) Raw data from liquid chromatography tandem mass spectrometry was subjected to trimming and analysis by first removing proteins with < 2 unique peptides. Spectral peptide peaks were aligned with Skyline software and peptide spectral matches (PSMs) were normalized to total PSMs prior to trimming. Finally, proteins found in < 75% of at least one lipoprotein fraction were excluded. (**B**) Principal components (PC) analysis was performed on each lipoprotein fraction (LDL in green, Lp(a) in red and VLDL in purple) using the trimmed data set and presented as a score plot for PC1 against PC2. (**C**) Normalized PSMs from each protein were transformed to a Z-score to demonstrate their relative abundance in each lipoprotein fraction. Lipoprotein fractions and proteins were grouped using Euclidean hierarchical cluster analysis and displayed in a heatmap (left panel).

We performed LC–MS/MS proteomics to determine isolated lipoprotein fraction enrichment based on their protein cargo. A complete list of identified proteins from each patients’ lipoprotein fractions and their PSMs are presented online on the PRIDE database^30^. The data analysis workflow is presented in Fig. 3A. Briefly, protein identifications with < 2 unique peptides were excluded from further analyses. Each protein’s PSM was normalized to the total observed PSMs in each sample. In total, we identified 112 proteins among the 4 fractions. Some proteins were only detected in a single sample (e.g., 1 out of the 7 LDL samples), thus we did not consider them characteristic to that or any other fraction. Protein identifications were filtered to include only proteins present in > 75% of samples from at least one lipoprotein compartment. Using this criterion, we identified 61 proteins in total.

Because Lp(a) density varies depending on the LPA isoform present, but falls between that of small dense LDL and HDL^22^, we compared the relative quantity of LPA in each sample from each lipoprotein fraction (Supplemental Fig. 3A). Lp(a) samples contained 93.6% of LPA detected in all samples. HDL fractions contained LPA in only one sample and was 0.81% of the total LPA detected. LDL samples contained 5.58% of all LPA and was present in 5 out of 7 samples. LPA was not detected in any VLDL samples. As expected, APOB was most abundant in Lp(a), LDL, and VLDL fractions, and least abundant in HDL (Supplemental Fig. 3B).

The relative abundance of each protein in each isolated lipoprotein fraction is represented in Supplemental Fig. 3C–E. APOB was the most abundant protein detected in Lp(a), LDL and VLDL (Supplemental Figs. 3C, E and F). LPA was the second most abundant protein in Lp(a) fractions.

The primary drivers of variance among samples were identified by principal components analyses (PCA). As expected, all lipoprotein classes clustered among themselves; HDL displayed a stark separation from Lp(a), LDL and VLDL (Supplemental Fig. 4). Because HDL was isolated with an independent method which may account for a batch effect, we performed a PCA excluding the HDL proteome. Like the PCA including all lipoprotein fractions, we identified lipoprotein fractions clustering tightly together apart from LDL which demonstrates the highest degree of variance (Fig. 3B). Hierarchal clustering of all lipoprotein fractions by their proteome using complete Euclidean distance analyses grouped each sample into its respective lipoprotein (Fig. 3C columns) mathematically confirming the successful isolation of lipoprotein classes.

### Small-volume isolation yields biologically active Lp(a) suitable for translational research

**Figure 4:**
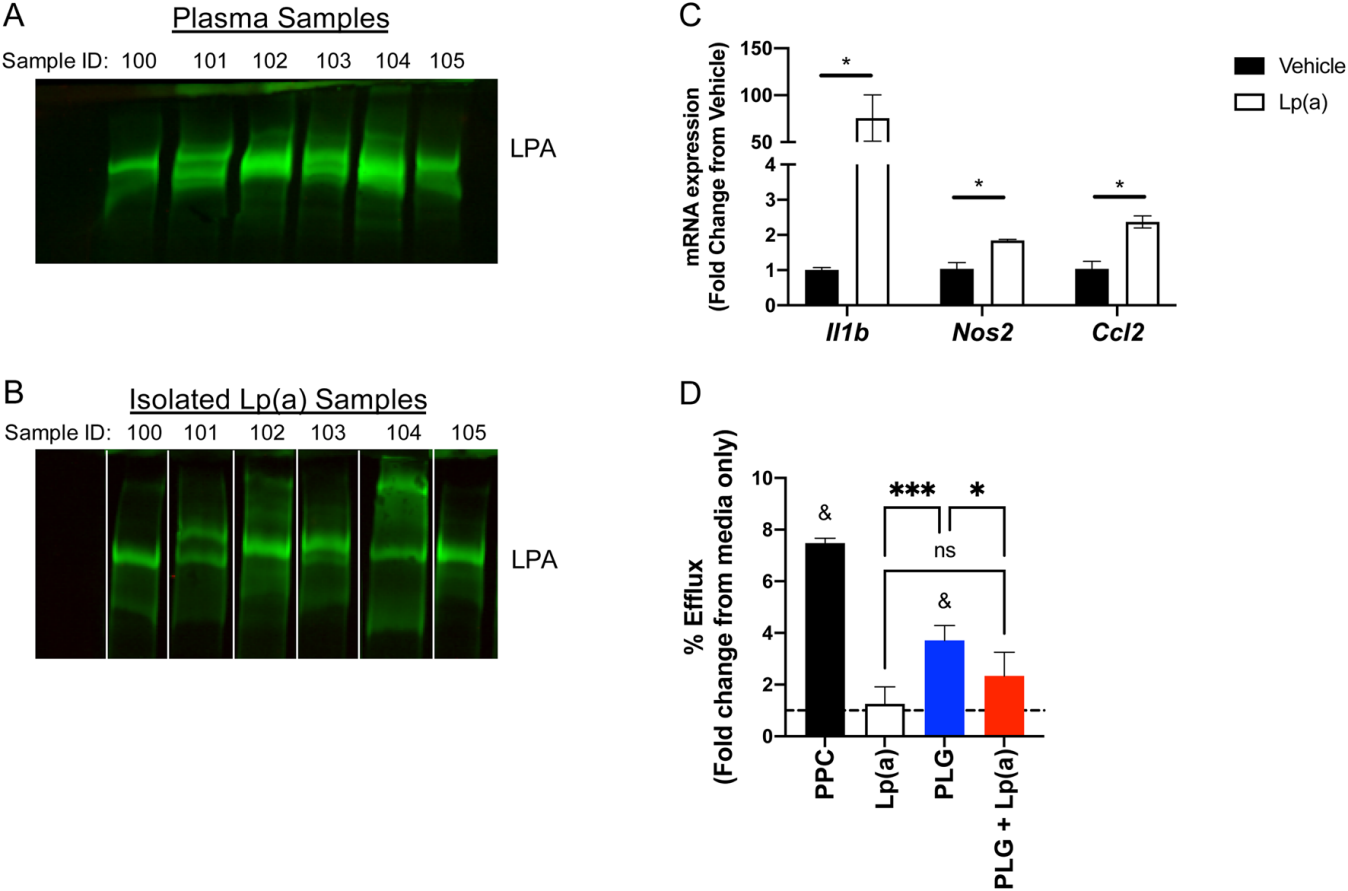
Small-volume isolation yields biologically active Lp(a) suitable for translational research. (**A**) Western blot using whole plasma from subjects with elevated Lp(a). LPA (green) is visualized above 460kD. The uncropped gel is presented in Supplemental Fig. 5. (**B)** Western blot was performed using Lp(a) isolated with our small-volume technique and LPA (green) visualized above 460kD. The original gel was cropped and lanes rearranged to match whole plasma presented in A. The unedited and uncropped gel is presented in Supplemental Fig. 6. (**C**) C57BL6/J mice were injected intraperitoneally with 3 mL thioglycolate broth (3%) and macrophages were collected from the peritoneum 4 days later. Macrophages were allowed to adhere to 6-well plates overnight and then treated for 24 h with DMEM containing 2% serum from Ldlr^-/-^ mice fed high-fat diet for 12 weeks to generate foam cells. Macrophage foam cells were treated with vehicle (black bars) or Lp(a) (15µg/mL; white bars) for 4 h. *Il1b*, *Nos2*, and *Ccl2* gene expression was determined via qRT-PCR as described in Materials and Methods. Lp(a) was derived from our small-volume isolation method and performed using Lp(a) from 4 separate subjects. Unpaired students t-test was used to identify differences between vehicle and Lp(a) treatment. *P < 0.05. (D) HEK293T cells were transfected with wildtype ABCA1 and radiolabeled with [^3^H]cholesterol overnight. Radiolabeled cells were treated for 4 h with pooled plasma control (black bar), isolated Lp(a) (15µg/mL; white bar) from 4 separate subjects, plasminogen (5µg/mL; blue bar), and pre-treatment of Lp(a) (15µg/mL) for 10-min followed by plasminogen (5µg/mL; red bar). Cholesterol efflux to the cholesterol acceptors was calculated as described in Materials and Methods and percent efflux (% efflux) is presented as fold change over radiolabeled cells treated with media only. One-way ANOVA with Tukey’s multiple comparisons test was used to identify differences among treatment groups. *P < 0.05; ***P < 0.001. One sample t and Wilcoxon test was used to determine differences between treatment and media only control. ^&^*P* < 0.05. Figures 4C and D were generated using GraphPad Prism version 9.3.1 for macOS, GraphPad Software, San Diego, California USA, www.graphpad.com.

To demonstrate that Lp(a) isolated using our small-volume method is suitable for translational research we evaluated for compositional and functional integrity. We first performed western blot analysis using whole plasma from subjects with elevated Lp(a) to evaluate LPA protein isoforms and identified various LPA isoforms among the subjects tested (Fig. 4A). Western blotting on Lp(a) isolated identified the same isoforms present in whole plasma samples (Fig. 4B).

Whole plasma from subjects with elevated Lp(a) was previously frozen; thus, to demonstrate that our isolated Lp(a) is active, we treated murine peritoneal macrophage foam cells with Lp(a) and measured inflammatory gene expression using qRT-PCR. Treatment of macrophage foam cells with Lp(a) induced 74.6-fold increase in *Il1b* (*P* = 0.04), a 1.79-fold increase in *Nos2* (*P* = 0.01), and a 2.28-fold increase in *Ccl2* (*P* = 0.008) mRNA expression compared to vehicle treated controls (Fig. 4C).

To test whether our small-volume Lp(a) fractions inhibit cholesterol efflux to plasminogen we performed cholesterol efflux assays on radiolabeled ABCA1 expressing HEK293T cells that demonstrated 7.48-fold increased ABCA1-dependent efflux when pooled plasma control was the cholesterol acceptor compared to media only (*P* < 0.0001). Aligned with previous reports^9^, Lp(a) did not elicit cholesterol efflux; however, it inhibited plasminogen mediated sterol efflux by 37.1% (Fig. 4D; 3.71-fold versus 2.34-fold for plasminogen compared to plasminogen and Lp(a) treatments, respectively; *P* = 0.046).

## Discussion

We developed an isolation method needing only 0.4 mL of plasma to collect Lp(a) for compositional and functional studies. Using this method, we isolated lipoprotein fractions from plasma in a clinical cohort consisting of patients presenting to our clinic with plasma Lp(a) ≥ 50 mg/dL. The addition of a dialysis step at the end of this method allows for tryptic digestion and LC–MS/MS proteomic analyses, which further confirmed enrichment and their diverse proteome. Finally, we demonstrate that the Lp(a) isolated from frozen plasma is biologically active, as it increases mRNA expression of inflammatory genes *Il1b*, *Nos2*, and *Ccl2*, and inhibits ABCA1-dependent efflux to plasminogen in vitro. Application of this technique should simplify and encourage clinical research into Lp(a) biology by reducing the plasma volume requirement.

Lp(a) is an independent risk factor for CAD, with 20–30% of the population having plasma Lp(a) levels ≥ 30 mg/dL. Statins are ineffective at decreasing Lp(a) levels in plasma^13–15^; instead, more challenging therapies are required for its reduction. These include *LPA* gene silencing^31–34^, lipoprotein apheresis^35^, or PCSK9 inhibition^35,36^. Isolation of plasma Lp(a) is a critical step in studying its biology, but previous isolation methods require significant amounts of starting material that ranges from 4 to 10 mL of plasma^22,23,37^. One commonly used technique to isolate Lp(a) requires ~ 4 mL of plasma in Optiprep™, a non-ionic density gradient medium of density 1.32 g/mL^37^; however, decreasing the starting plasma volume leads to a failure to resolve the Lp(a) compartment from LDL. In 2011, von Zychlinski et al. described using a combination of density-gradient ultra-centrifugation followed by size-exclusion fast-performance liquid chromatography (SE-FPLC) that required only 4 mL of plasma^22^. Their method utilizes KBr at a density of 1.063 mg/mL during the initial separation of VLDL/LDL from Lp(a)/HDL followed by adjusting the latter plasma fraction density to 1.09 mg/mL. This latter protocol discards VLDL and LDL and relies upon SE-FPLC to separate Lp(a) and HDL. Diluted fractions from SE-FPLC are combined and used for proteomic analyses, but the total yield was not reported and thus whether other applications can be used with this method remains unclear. von Zychlinski et al. would modify this protocol later in 2014 to isolate VLDL, LDL and HDL; however, they used plasma from patients lacking Lp(a)^38^. Our method utilizes a similar two-step density-gradient ultracentrifugation but modifies the density from previous methods, with the first step at a density of 1.066 g/mL generating a component consisting of VLDL/LDL and another consisting of Lp(a)/HDL. Prior to the second spin, VLDL/LDL densities are adjusted to 1.027 g/mL and Lp(a)/HDL density is adjusted to 1.082 g/mL to further separate VLDL from LDL and Lp(a) from HDL. The yield of Lp(a) strongly correlated with the concentration of plasma Lp(a) and averaged 61.5 µg. This yield provided sufficient material for several experimental applications including LC-MS/MS proteomic analysis, immunoblotting, lipid gel analysis, in vitro gene expression assays and cholesterol efflux assays. Thus, compared to previously reported protocols, our method requires far less plasma and less operator time (ultracentrifugation and dialysis as compared to manually running each sample through FPLC). Due to the time constraint required for execution of this small-volume isolation we recognize that this method is not suitable for high-throughput applications. On the other hand, others have successfully utilized a similar sequential density-gradient ultracentrifugation technique to isolate HDL in cohort sizes ranging from ~ 1,000 to 3,000 subjects^39,40^; thus, this method is suitable for medium-throughput applications.

We enlisted subjects with Lp(a) ≥ 66 mg/dL in this study for two reasons: (1) previous reports that incident rate ratios for risk of major adverse cardiac events for subjects with Lp(a) ≥ 50 mg/dL is 1.42 compared to subjects with Lp(a) ≤ 10 mg/dL^41^, thus these subjects are representative of patients with heightened risk of major adverse cardiac events; and (2) sufficient Lp(a) yields are necessary to perform the biological assays presented in this study. To test whether this novel small-volume isolation method is suitable to isolate Lp(a) from patients with plasma Lp(a) < 50 mg/dL, we isolated Lp(a) from two additional subjects presenting to our clinical cohort with plasma Lp(a) of 30 mg/dL and 11 mg/dL. Our isolation resulted in similar percent yields as subjects with plasma Lp(a) ≥ 66 mg/dL, but the actual Lp(a) yields were 13.7 and 5.6µg in subjects with 30 mg/dL and 11 mg/dL plasma Lp(a), respectively. Thus, it is feasible to use this small-volume method for subjects with Lp(a) < 50 mg/dL; however, the low Lp(a) yields render it difficult to perform multiple biological assays. Thus, these latter subjects were excluded from the current study.

Our primary concern for small-volume lipoprotein fraction isolation was fraction enrichment. Lp(a) density (1.05–1.09 g/mL) overlaps with that of LDL (1.019–1.063 g/mL) and HDL (1.063–1.21 g/mL)^42^, which renders its isolation via density-gradient ultracentrifugation problematic. On average, 6.19% of the total LPA was recovered in LDL compared to 93.8% in the Lp(a) fractions as determined by Western blotting. Similarly, the highly sensitive LC/MS-MS analysis revealed trace amounts of LPA in 6 out of 9 LDL samples accounting for 5.58% of total LPA compared with the Lp(a) samples which contained 93.6% of the total LPA. As a result, this method may not be suitable for isolating LDL in patients with elevated Lp(a). With regard to our HDL isolation, only one out of eight HDL samples had detectable LPA representing 0.81% of total LPA suggesting that the established HDL isolation protocol is suitable for the separation of an enriched HDL even in patients with high circulating Lp(a). HDL separates distinctly on the PCA analysis and spatial clustering compared to the rest of the lipoprotein fractions and this may be due to a batch effect as the HDL samples in this study derive from a separate isolation than Lp(a), LDL and VLDL. Thus, we performed a PCA analysis that excluded the HDL proteome and show that samples from each fraction cluster together. Our method’s major limitation is that while Lp(a), LDL, and VLDL are obtained from one isolation, HDL requires a subsequent low-volume isolation of its own. Additionally, while useful for characterizing the lipoprotein protein cargo our total PSM normalization method is only semi-quantitative; thus, peptide abundance presented in this study may not reflect the absolute stoichiometry of each lipoprotein protein cargo. We used FPLC to visualize each lipoprotein class’s cholesterol concentration curves and identified that Lp(a) isolated from a subject with two different size-isoforms demonstrated two unique peaks—one just prior to the start of the LDL curve and another larger peak between LDL and HDL. Thus, it is feasible that size-exclusion chromatography following small-volume isolation of Lp(a) may distinguish between different Lp(a) isoforms.

We next tested whether our isolated Lp(a) was suitable for translational research by employing common laboratory assays relevant to Lp(a) biology including in vitro gene expression assays and measuring cholesterol efflux. Our results show that isolated Lp(a) was capable of inducing mRNA expression of the inflammatory genes *Il1b*, *Nos2* and *Ccl2* in primary murine macrophage foam cells. These results align with previous reports demonstrating Lp(a) is capable of increasing MCP-1 (encoded by the gene *CCL2*)^43^. Lp(a) was also shown to increase IL-6 in human monocytes ^44^, a well-characterized inflammatory cytokine downstream of IL-1β^45,46^. On the other hand, a previous study demonstrated that oxidized Lp(a) reduces *Nos2* mRNA and NOS2 protein in the J774A.1 murine macrophage cell line pretreated with interferon gamma and LPS^47^. It should be noted that our results do not contradict these reports as our experiments used primary murine macrophage foam cells generated from naïve peritoneal macrophages incubated for 24 h with serum from *Ldlr*^-/-^ mice on Western type diet for 12 weeks. Additionally, we did not measure the oxidation level of the Lp(a) isolated with our method.

The LPA gene is a duplication of the gene encoding plasminogen (PLG); thus, the two bear structural homology in the form of 10 kringle domains that differ in their sequence^48,49^. Our lab has previously shown that plasminogen promotes ABCA1-dependent cholesterol efflux^9^. We have also demonstrated, using previously established large-volume Lp(a) isolation techniques, that Lp(a) inhibits cholesterol efflux mediated by plasminogen, but not APOA1^9,10^.

We developed a novel small-volume method for the isolation of Lp(a) from 0.4 mL of plasma with yields sufficient to perform in-depth analytics via mass spectrometry as well as a wide range of experimental applications to study composition and function of Lp(a). Thus, the ability for research laboratories to isolate Lp(a) from low plasma volumes will expand the focus on Lp(a) in cohort studies with limited availability of stored samples.

## Supplementary Information

Supplementary Information.

## Abbreviations

Lp(a): Lipoprotein(a)
LPA: Apolipoprotein (a)
APOB: Apolipoprotein B
FPLC: Size exclusion-fast flow liquid chromatography
CAD: Coronary artery disease
PCA: Principal component analysis

## Human genes

*LPA*: Lipoprotein(a)
*PLG*: Plasminogen
