# Supplementary material for: A method for lipoprotein (a) Isolation from a small volume of plasma with applications for clinical research
Source: Sci Rep. 2022 Jun 1;12:9138. doi: 10.1038/s41598-022-13040-4 (PMC9160242; doi:10.1038/s41598-022-13040-4)
Supplement: Supplementary file 1 — Supplementary Information. [file 41598_2022_13040_MOESM1_ESM.docx]

**Supplemental Material**

| **Low Lp(a) Subject** | **Plasma Lp(a) (mg/dL)** | **Expected yield (μg)** | **Actual Lp(a) yield (μg)** | **Percent Yield (%)** |
| --- | --- | --- | --- | --- |
| 1 | 30 | 120 | 13.7 | 11.4 |
| 2 | 11 | 44 | 5.6 | 12.7 |

**Supplemental Table 1. Lower limit of Lp(a) isolation in subjects with Lp(a) < 50mg/dL.** Small-volume isolation was performed in two subjects presenting with Lp(a) < 50mg/dL. Plasma Lp(a) concentrations (mg/dL) and yield results are presented.


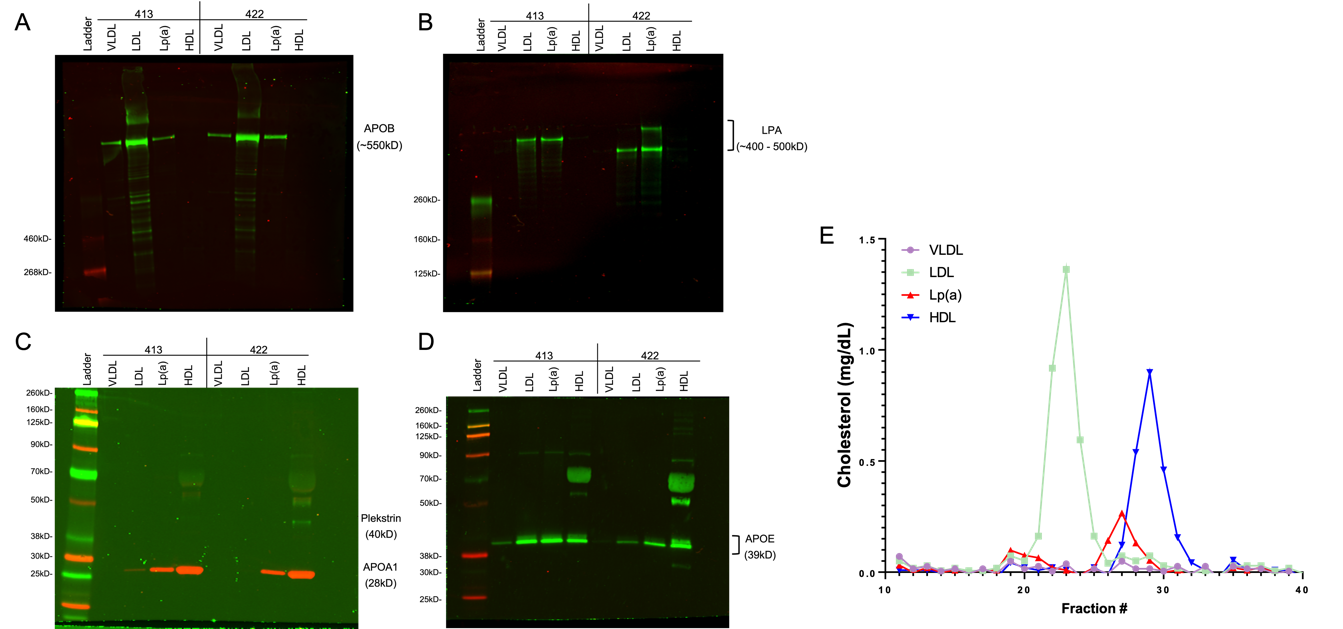


**Supplemental Figure 1. Apolipoprotein expression isolated lipoprotein fractions and cholesterol distribution.** VLDL, LDL, Lp(a), and HDL was isolated as described in Materials and Methods. Western blot was performed with 1μL of each isolated fraction and visualized for A) APOB, B) LPA, C) PLEK and APOA1, and D) APOE. E) Each isolated fraction was subjected to size-exclusion chromatography (FPLC). Total cholesterol was quantified in each FPLC fraction using Pointe Scientific Cholesterol Liquid Reagent Set. VLDL (purple), LDL (green), Lp(a) (red), and HDL (blue) cholesterol distribution curves are displayed in mg/dL.


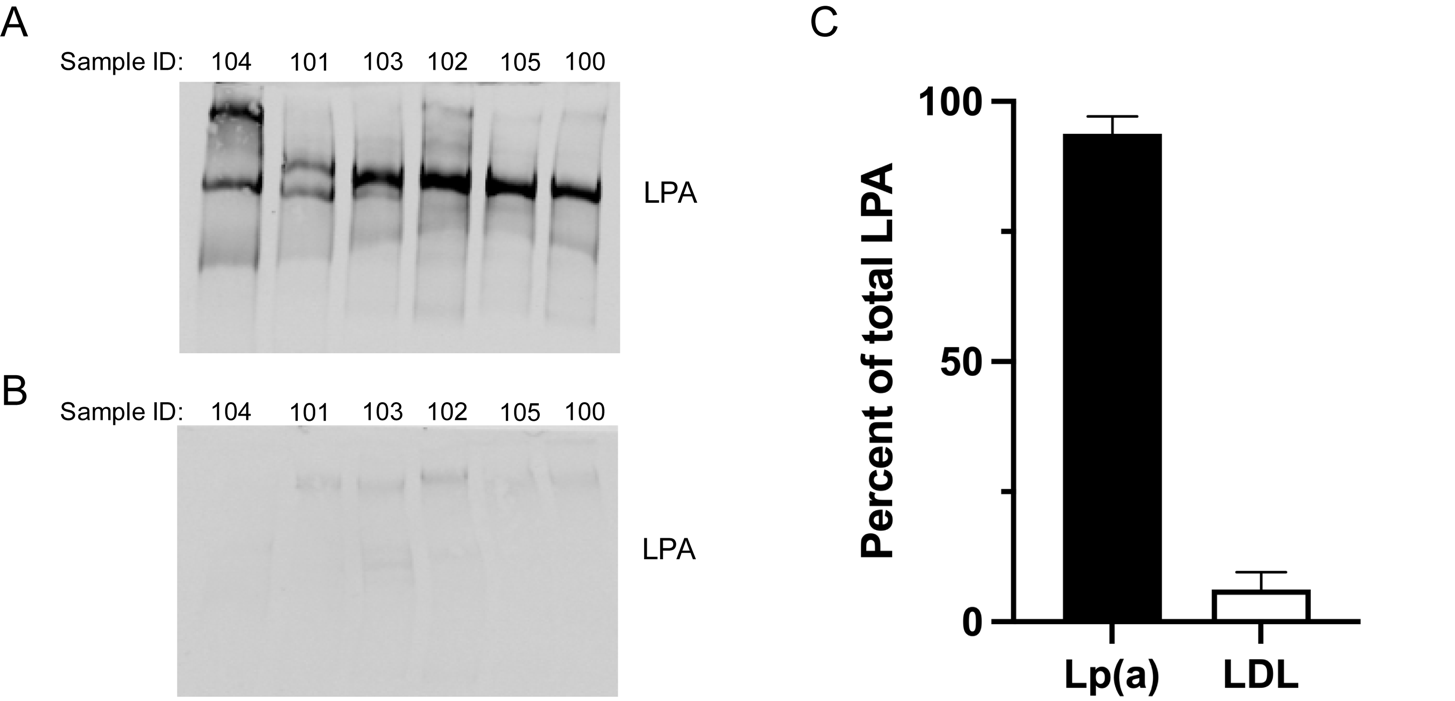


**Supplemental Figure 2. Isolated Lp(a) fractions are enriched in LPA compared to isolated LDL.** Western blot was performed using 10μg of A) Lp(a) and B) LDL fractions isolated with our small-volume technique. LPA is visualized above 460kD and both blots were analyzed simultaneously. Uncropped images of the membranes are presented in Supplemental Figure 6. C) Total LPA signal was quantified for each sample and each fraction’s percent of total LPA presented.

**Supplemental Figure 3. Summary of relative protein abundance in isolated lipoprotein fractions.** A) LPA total normalized peptide spectrum matches (PSMs) from each lipoprotein fraction. B) APOB total normalized PSMs from each lipoprotein fraction sample. C – F) Proteins were normalized to each sample’s total PSMs and sorted by abundance.

**Supplemental Figure 4. Principal components analysis (PCA) of Lp(a), HDL, LDL, and VLDL proteomes.** Principal components (PC) analysis was performed on each lipoprotein fraction (HDL in blue, LDL in green, Lp(a) in red and VLDL in purple) using the trimmed data set and presented as a score plot for PC1 against PC2.


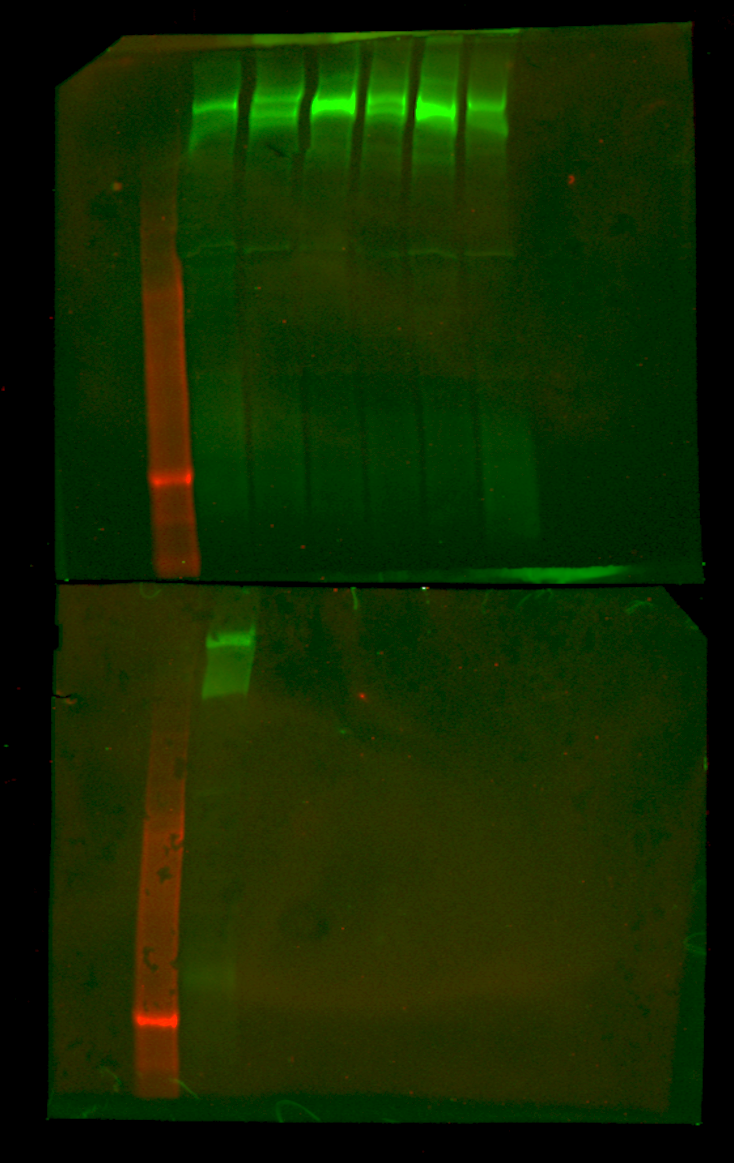


**460 kDa**

**Supplemental Figure 5.** Western blot was performed using plasma (1μL). LPA is visualized above 460kD and both blots were analyzed with a LICOR Odyssey CLx Near-Infrared Imaging System with the same scan.


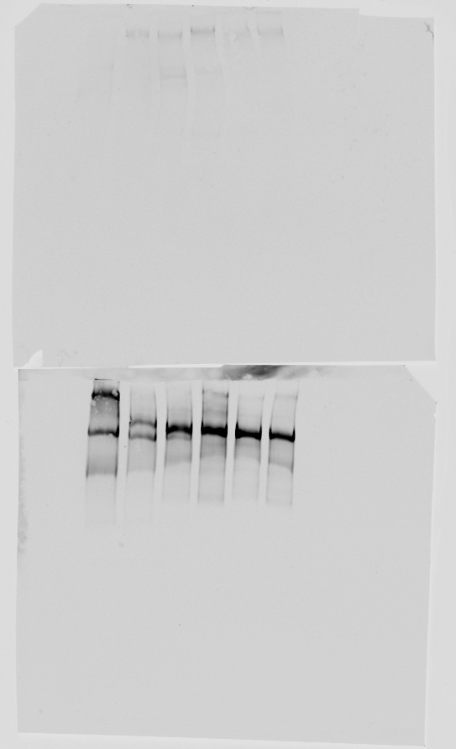


**Supplemental Figure 6.** Western blot was performed using LDL fractions (top membrane) and Lp(a) (bottom membrane) isolated with our small-volume technique. Lipoprotein concentration was determined with BCA protein assay and 10μg of total protein was loaded to each well. LPA is visualized above 460kD and both blots were analyzed with a LICOR Odyssey CLx Near-Infrared Imaging System with the same scan.
